# Supramolecular Optimization of Sensory Function of a Hemicurcuminoid through Its Incorporation into Phospholipid and Polymeric Polydiacetylenic Vesicles: Experimental and Computational Insight

**DOI:** 10.3390/polym15030714

**Published:** 2023-01-31

**Authors:** Bulat S. Akhmadeev, Olga O. Retyunskaya, Sergey N. Podyachev, Sergey A. Katsyuba, Aidar T. Gubaidullin, Svetlana N. Sudakova, Victor V. Syakaev, Vasily M. Babaev, Oleg G. Sinyashin, Asiya R. Mustafina

**Affiliations:** 1 Arbuzov Institute of Organic and Physical Chemistry, FRC Kazan Scientific Center of RAS, 8 Arbuzov St., 420088 Kazan, Russia; 2 Department of Organic and Medicinal Chemistry, Kazan (Volga region) Federal University, Kremlyovskaya Str., 18, 420008 Kazan, Russia

**Keywords:** polydiacetylene, hemicurcuminoids, sensor

## Abstract

This work presents the synthesis of a new representative of hemicurcuminoids with a nonyloxy substituent (**HCur**) as a fluorescent amphiphilic structural element of vesicular aggregates based on phosphatidylcholine (PC), phosphatidylserine (PS), and 10,12-pentacosadiynoic acid (PCDA). Both X-ray diffraction analysis of the single crystal and ^1^H NMR spectra of **HCur** in organic solvents indicate the predominance of the enol-tautomer of **HCur**. DFT calculations show the predominance of the enol tautomer **HCur** in supramolecular assemblies with PC, PS, and PCDA molecules. The results of the molecular modeling show that **HCur** molecules are surrounded by PC and PS with a rather weak exposure to water molecules, while an exposure of **HCur** molecules to water is enhanced under its supramolecular assembly with PCDA molecules. This is in good agreement with the higher loading of **HCur** into PC(PS) vesicles compared to PCDA vesicles converted into polydiacetylene (PDA) ones by photopolymerization. **HCur** molecules incorporated into **HCur**-PDA vesicles exhibit greater planarity distortion and hydration effect in comparison with **HCur**-PC(PS) ones. **HCur**-PDA is presented as a dual fluorescence-chromatic nanosensor responsive to a change in pH within 7.5–9.5, heavy metal ions and polylysine, and the concentration-dependent fluorescent response is more sensitive than the chromatic one. Thus, the fluorescent response of **HCur**-PDA allows for the distinguishing between Cd^2+^ and Pb^2+^ ions in the concentration range 0–0.01 mM, while the chromatic response allows for the selective sensing of Pb^2+^ over Cd^2+^ ions at their concentrations above 0.03 mM.

## 1. Introduction

The supramolecular assembly of different amphiphilic molecules allows for the incorporating of sensors into vesicular aggregates with high colloid stability and biocompatibility, which provides a good basis for creating sensory systems [1,2,3,4,5]. The uses of colorimetric and luminescence techniques for sensing are particularly attractive since they can facilitate the naked-eye or simple spectroscopic detection of biomolecules or water-soluble toxicants [6,7,8,9,10,11]. The development of sensory systems on the basis of supramolecular assemblies requires structure optimization on both molecular and supramolecular levels. 

**Scheme 1:**
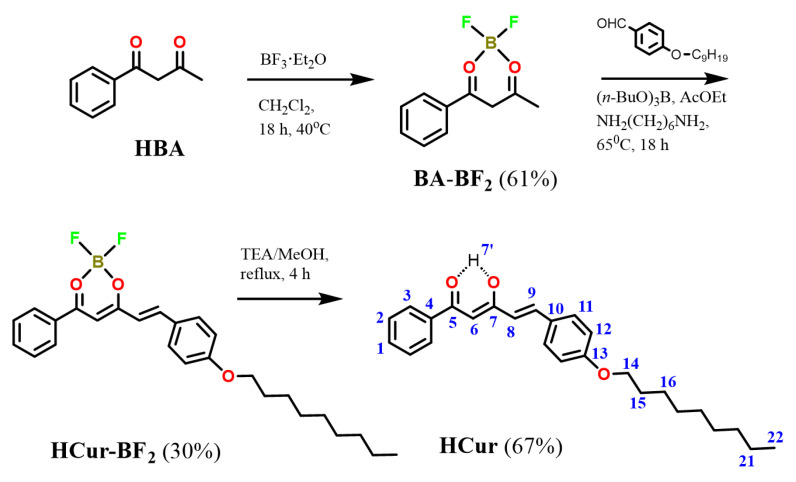
Synthetic sequence for the preparation of **HCur**.

Curcumine and curcuminoid derivatives provide good basis for the development of sensors [12,13,14,15,16,17]. Moreover, synthetic modifications of curcuminoids and hemicurcuminoids allow an embedding of different functional groups into their molecules [18,19]. The resulting hemicurcumine **HCur** (also shown in Scheme 1) will be represented as a potential sensor for the fluorescent monitoring of heavy metal ions and polyaminoacids. To overcome the poor water solubility of **HCur,** it has been incorporated into water-dispersible hydrophilic vesicular aggregates such as phospholipid vesicles and polydiacetylenic polymeric vesicular aggregates (PDAs), which are already documented as convenient nanoplatforms for the incorporation of luminophores, both organic [20] and complexed with metal ions [21,22,23].

It is well known that both the conformation and the electronic structure of dye molecules inserted into phospholipid vesicles are greatly influenced by their environment, which causes the sensitivity of dye molecules’ phase transitions of phospholipid bilayers [24]. Moreover, both the conformational flexibility and hydrophobic or hydrophilic environment of a dye molecule have a major influence on its sensory function. Therefore, the inclusion of **HCur** molecules into mixed vesicular aggregates having both surface groups capable of pH-dependent ionization and a hydrophobic bilayer would provide a convenient basis for designing sensor systems focusing on the binding of both heavy metal ions and polylysine.

It is also worth noting that 10,12-pentacosadiynoic acid molecules are able to form polymeric polydiacetylene (PDA) nanoparticles after their self-organization into bilayers and subsequent polymerization [25,26]. Moreover, the relationship between the protonation/deprotonation of the surface-exposed carboxylic groups of PDA and ordering/disordering within their hydrophobic ene-yne conjugation backbone [27,28,29,30] is the already-documented reason for the colorimetric response to the binding event [31,32,33]. The interplay between fluorescent and colorimetric responses of **HCur** molecules and PDA vesicles, respectively, will also be demonstrated as a tool to alter the sensory functions of mixed **HCur**-PDA vesicles. The present work is aimed at using the assembly of **HCur** molecules with phospholipids or 10,12-pentacosadiynoic acid (a monomeric unit of PDA polymer vesicles) as a tool for tuning the conformation and electronic structure of **HCur** for the modification of its sensory function.

## 2. Experimental Section

### 2.1. Reagents and Materials 

AcOEt (Acros Organics) was distilled over P_2_O_5_. DMSO-*d*_6_ (99.5% isotopic purity) from Aldrich was used for NMR spectroscopy. Methanol (99.9%), 1-benzoylacetone (**HBA**), 4-hydroxybenzaldehyde, triethylamine (TEA), BF_3_·Et_2_O (48%), and 1,6-diaminohexane (Acros Organics) were used as commercially received without further purification.

Compounds 10,12-pentacosadiynoic acid, L-a-Phosphatidylcholine (P3644, M_av_ = 776 g/mol),1,2-Diacyl-sn-glycero-3-phospho-L-serine (P7769, M_av_ = 790 g/mol) were received from Sigma Aldrich.

**Synthesis:** The synthetic routes, structural formulae and numbering of atoms of the investigated compounds are shown in Scheme 1. The 4-nonyloxybenzaldehyde and benzoylacetone-difluoroboron (**BA-BF_2_**) were obtained as described in the literature [34,35]. ^1^H and ^13^C chemical shifts and spin-spin coupling constants observed for synthesized hemicurcuminoids **HCur-BF_2_** and **HCur** are presented in Table S1. **HCur** was characterized by high resolution mass spectrometry (HRMS) data (Figure S1).

Synthesis of **HCur** was performed through the preliminary synthesis of **HCur-BF_2_** (the detailed synthetic procedure is in the Supplementary Materials) followed by its hydrolysis with the production of **HCur** (the detailed procedure is in the Supplementary Materials).

### 2.2. Synthesis of the Mixed Vesicles

**HCur-PC(PS).** The L-α-Phosphatidylcholine (PC), 1,2-Diacyl-sn-glycero-3-phospho-L-serine (PS) and **HCur** were dissolved in chloroform. Aliquots of **HCur** (0.314 mL, 0.26 mM) and PC (2.0 mL, 2.58 mM) or PS (2 mL, 2.53 mM) were mixed and evaporated at 40 °C and ≈450 mbar. De-ionized water (16 mL) was added to the thin film, and the resulting solution (0.32 mM PC/PS with 0.05 mM of **HCur**) was sonicated in an ultrasonic bath for 4 min at 25 °C. Vesicles **HCur**-PC and **HCur**-PS were used as is after the synthesis.

**HCur-PDA.** The synthesis of **HCur**:PDAs were in accordance with the previously published procedure [36]. The monomeric 10,12-tricosadiynoic acid was dissolved in chloroform and filtered by using a 0.45 μm nylon filter to remove polymerized particles. Aliquots of 10,12-PCDA (1.87 mL, 4.27 mM) and **HCur** (0.314 mL, 0.26 mM) were mixed and evaporated at 40 °C and ≈450 mbar. De-ionized water (16 mL) was added to the thin film, and the resulting solution (0.5 mM PCDA with 0.05 mM of **HCur**) was sonicated in an ultrasonic bath for 4 min at 60 °C. Vesicles were stored at 4 °C within 24 h, and then were polymerized in a Petri dish using UV Crosslinker Bio-link 254 for 60 s.

The molar ratios of **HCur**:PC, **HCur**:PS and **HCur**:PDA were determined through the spectral analysis of the residual amounts of **HCur** after the thin film step according to the procedure described in Supplementary Materials. The molar ratios were calculated through Equation (1) represented in the Supplementary Materials

### 2.3. Methods

The detailed descriptions of common methods such as C, H microanalysis, mass spectra, high resolution mass spectra, dynamic light scattering (DLS), conditions and equipment for electronic absorption and fluorescence spectra measurements, and pH-measurements are presented in the Supplementary Materials.

The equipment for collecting of X-ray diffraction data is described in the Supplementary Materials. The collection and treatment of the data was conducted on the basis of well-known techniques and programs [37,38,39,40,41]. Crystallographic data (excluding structure factors) for the investigated structure **3** has been deposited in the Cambridge Crystallographic Data Centre as supplementary publication no. CCDC 2213569. Copies of the data can be obtained free of charge upon application to the CCDC (12 Union Road, Cambridge CB2 1EZ UK. Fax: (internat.) +44-1223/336-033; E-mail: deposit@ccdc.cam.ac.uk).

### 2.4. Computations

The thermodynamically correct structural ensembles for the conformers of each possible tautomer of **HCur** were generated with the use of CREST (short for Conformer-Rotamer Ensemble Sampling Tool) program [42]. Free energies of the generated species in solutions were calculated with the recently published CENSO protocol of Grimme et al. [43], designed for the evaluation of structure ensembles containing non-rigid molecules. The geometries were optimized with the r^2^SCAN-3c [44] composite density functional in their respective solutions using the DCOSMO-RS [45] implicit solvation model, whereby interactions with the environment are already considered from the beginning. Solvation contributions δ*G*_solv_ to the free energy at 298.15 K were obtained with COSMO-RS [46,47] using the *BP_TZVP_C30_1601.ctd* parameterization in combination with the energy and density of the high-level single-point calculation. The thermostatistical contributions to the free energy were obtained by single-point hessian (SPH) calculations [48] within the framework of the modified rigid-rotor-harmonic-oscillator statistical treatment (Δ*G*_mRRHO_) [49,50] at the GFN2-xTB [51] /ALPB [52] level, where GFN2-xTB is the robust and fast semi-empirical quantum chemical method and ALPB is the robust and efficient implicit solvation model for fast semiempirical methods. The final free energies were obtained from Δ*G* = Δ*E*(r^2^SCAN-3c) + Δ*G*_mRRHO_(GFN2-xTB/ALPB) + Δδ*G*_solv_(COSMO-RS).

The most stable conformers of each tautomer, revealed on the basis of computed Δ*G* values, were used in the computation of UV-Vis spectra. Time-dependent density functional response theory (TD-DFT) [53,54,55] has been employed to compute the vertical excitation energy (i.e., absorption wavelengths) and oscillator strength on the ground state geometries optimized within the framework of the CENSO protocol as described above. For this purpose, the PBE0 function [56] in combination with the Ahlrichs’ triple-ζ def2-TZVP AO basis set [57,58,59] was used. 

The same conformers were further used as starting structures to generate clusters of these species explicitly solvated by PC or PCDA molecules. Clusters are generated by the *Quantum Cluster Growth* (QCG) algorithm [60,61] which, in short, adds solvent molecules around the solute at energetically favorable positions using an intermolecular force field docking algorithm (xTB-IFF) [20]. The cluster-generation step is followed by molecular dynamics (MD) simulations with the use of the recently developed general force field GFN-FF [62]. Equilibrated snapshots from the trajectory are fully geometry-optimized at the GFN2-xTB level, forming an ensemble of low-energy clusters. The dynamical behavior of the clusters of the lowest free energy (i.e., including thermostatistical corrections) was further studied within the framework of the MD approach. MD trajectories of 1200 ps were carried out with the use of a generic all-atomic force field GFN-FF. All DFT and TD-DFT calculations were carried out using the Turbomole-7.5.1 program package [63].

## 3. Results and Discussion

### 3.1. Synthesis and Structure of Hemicurcuminoids

The synthesis of the hemicurcuminoid **HCur-BF_2_** was carried out by the condensing of 4-nonyloxy-benzaldehyde with the boric complex of benzoylacetone (**BA-BF_2_**) (Scheme 1). Aldol condensation and the subsequent destruction of the boron complex, achieved by its treatment of boiling in MeOH with the addition of TEA in the case of **HCur-BF_2_**, leads to the formation of target hemicurcuminoid (**HCur)**. The obtained compounds were characterized by the elemental analyses, NMR and MS techniques. According to ^1^H NMR data, the compound **HCur** in DMSO-*d_6_* solution at a concentration of 0.03 M is found in enol form (>99%).

**Figure 1:**
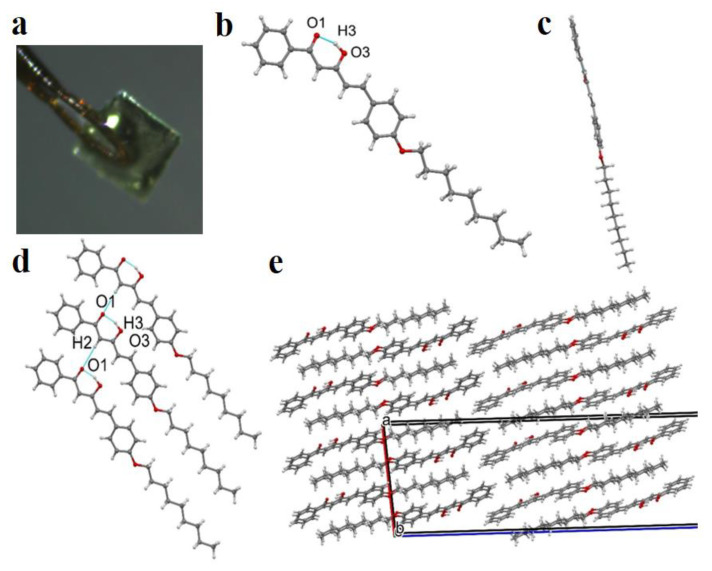
(**a**) Photo of the single crystal **of HCur**; (**b**,**c**) ORTEP view of two projections of the molecule in the crystal. Non-hydrogen atoms are represented by probability ellipsoids of thermal vibrations (*p* = 50%), hydrogen atoms by spheres of arbitrary radii. (**d**) O-H…O and C-H…O hydrogen bonds (blue dashed lines) in the crystal **3**; (**e**) molecular packing fragment in the crystal **3,** hydrogen atoms are not shown for clarity.

The structure of **HCur** was finally established by a single crystal X-ray crystallography of the separated crystal grown from the DMSO-*d*_6_ (Figure 1a). Compound **HCur** belongs to the monoclinic system, space group *C2/c* (Table S2). The crystal consists only of hemicurcuminoid **3** molecules, which are in enol form as well as in DMSO-*d_6_* solution. This form is stabilized by intramolecular hydrogen bonds between the hydrogen atom of the hydroxyl group and the oxygen atom of the carboxyl group [O3-H3···O1 (2.4736(15) Å] (Figure 1b). In general, the molecule has a flat elongated structure where all carbon atoms are practically in the same plane (Figure 1c). This fact can be explained by the conjugation effect in the molecule and the effect of packing. 

An intermolecular hydrogen bond was observed between the hydrogen atom of the hydroxy group and the oxygen atom of the carbonyl group [C2-H2···O1 (2.690(17) Å, symmetry code: x,−1+y,z] (Figure 1d). This hydrogen bond forms a chain structure along the *b*-axis. A molecular packing diagram of **HCur** is shown in Figure 1e and represents an antiparallel stacking of H-chains along the a-axis mainly through the van der Waals interactions. Although such packing leads to the absence of voids in the crystal, it does not lead to the densest packing, since the calculated packing factor of Kitaygorodsky is equal to 0.699, which is in the middle part of the range characteristic for crystals of organic compounds (0.65–0.75). At the same time, despite the planar conformation, these molecules turn out to be sufficiently labile for the formation of various types of crystal packings, as evidenced by our obtaining crystals of this compound of a different triclinic modification. Research on these crystals is ongoing.

### 3.2. Synthesis and Spectral Properties of PDA-HCur, PC-HCur, PS-HCur 

**Figure 2:**
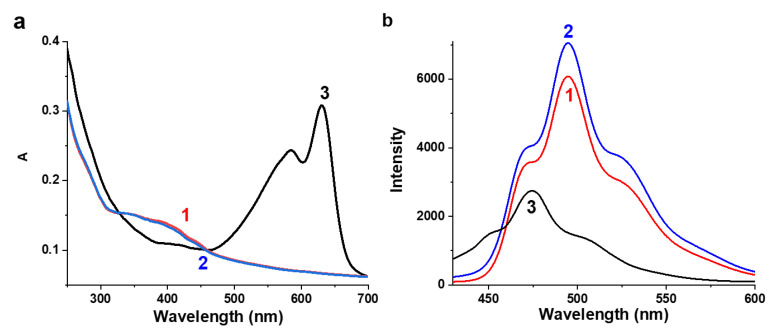
Electronic absorption (**a**) and emission spectra (**b**) of **HCur**-PC (C**_HCur_** = 0.041), **HCur**-PS (C**_HCur_** = 0.036) and **HCur**-PDA (C**_HCur_** = 0.011).

The amphiphilic nature of **HCur** (its structure is shown in Scheme 1) is the reason for its incorporation into the PC-, PS- and PCDA-based vesicles. The nonyl substituents of the **HCur** molecules provide the driving force of their incorporation into PC-, PS- and PCDA-based vesicles through the well-known hydrophobic effect, resulting in the mixed vesicles’ formation. The mixed vesicles were synthesized through the modified thin-film synthetic procedure described in detail in the Exp. Section. The synthesis was performed at various **HCur**:PCDA molar ratios, followed by analysis of residual amounts of **HCur** after the exposure of the mixed thin film (PCDA-**HCur**) to an aqueous solution under ultrasonication. The residual amounts of **HCur** were evaluated by spectrophotometry after their dissolution in chloroform (more details are in the Supplementary Materials, Figure S2). These results allow the optimization of the synthetic procedure and calculation of the **HCur**:PCDA molar ratio (1:45) in the mixed vesicles. A similar synthetic strategy was applied in the synthesis of the mixed PC- and PS-based vesicles. The analysis of the residual amounts of **HCur** also confirms its incorporation into the PC- and PS-based vesicular aggregates, although the calculated **HCur**:PC(PS) molar ratios are 1:12 and 1:14, correspondingly. The incorporation of **HCur** molecules into the phospholipid vesicles is manifested by the electronic absorption bands shown in Figure 2a. Contributing to the spectral profile of **HCur**-PDA is the intensive bands at 560–640 nm arising from the ene-yne conjugation backbone of the PDA nanoplatform, while the electronic absorption of **HCur** in **HCur**-PDA is blue-shifted vs. **HCur**-PC(PS) (Figure 2a). The poor photobleaching of **HCur** under the irradiation required for the photopolymerization (Figure S2) prerequisites the conversion of **HCur**-PCDA vesicles into **HCur**-PDA polymeric ones without the significant photodegradation of **HCur** molecules.

**Figure 3:**
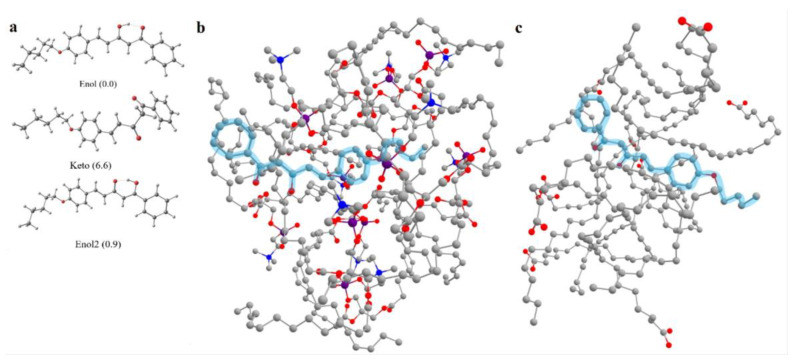
(**a**) Structures of the most energetically stable conformers of the simplified model of **HCur** and their relative Gibbs free energies (ΔG/kcal·mol^−1^, in parentheses) computed with the use of CENSO protocol for chloroform solution. (**b**,**c**) Optimized structures of cluster **HCur**-PC and **HCur**-PCDA obtained by MD simulation, where hydrogen atoms are omitted for clarity.

The electronic absorption and emission spectral profiles of **HCur** are greatly affected by the nature of the nanoplatform (Figure 2), although the represented spectra differ from those performed in chloroform and DMF solutions (Figure S3). The main difference is the increased intensity of the longer wavelength emission, which shifts the maximum from 471 nm in DMF to 494–500 nm in PC and PS vesicles, while the emission at 470–475 nm still remains as the shoulder in the spectra along with the appearance of the shoulder at ~570 nm (Figure 3b and Figure S3). The spectra of **HCur** incorporated into PDA vesicles have a maximum at ~480 nm, while the emission at ~510 and ~555 nm is manifested by the shoulders (Figure 2b). It is worth discussing the main factors affecting the emissive properties of **HCur** molecules in the mixed vesicles, since the properties are dependent on the nature of the nanoplatform. 

The keto-enol tautomeric equilibrium shift is the main factor influencing the luminescence of curcuminoid derivatives [64]. The computations were performed in order to reveal which tautomeric form of **HCur** is most favorable in their mixed aggregates with PC molecules. Three possible tautomers of the simplified model of **HCur** are shown in Figure 3. According to the free energy values computed for molecules implicitly solvated by dimethylformamide or chloroform, enol tautomer strongly dominates in both media. It should be noted that the same form is also found in single crystals of **HCur** (cf. Figure 1b and Figure 3a). UV-Vis spectra were simulated for the most stable conformers of each tautomer (Figure 3a), revealed on the basis of their computed free energies. The TD-DFT simulated spectrum of keto tautomer contains two strong bands in the spectral interval ~300–450 nm, while only one band at ~390 nm is registered in the UV-Vis spectra of solutions. The latter band is fairly well matched by the only strong band (~400 nm) in this interval, which is computationally predicted for enol and enol2 tautomers. The experimental absorption spectra of **HCur**-PC and **HCur**-PCDA in the spectral interval ~300–450 nm are very similar to the spectra of solutions discussed above, which strongly suggests that enol tautomers are the major forms of **HCur** in all systems under study. 

For the above reason, computational modeling of the structural arrangement of **HCur**-PC and **HCur**-PCDA aggregates was conducted for the single enol tautomer of **HCur** surrounded by (a) ten PC or (b) ten PCDA molecules in the aqueous environment modeled implicitly. According to our computations of system (a), Ph(C=O) moiety of **HCur** is pushed to the periphery of the cluster (Figure 3b) and almost completely immersed in the surrounding water shell. The molecule of the dye is situated in a rather narrow well, formed mainly by long hydrocarbon tails of PC. In contrast, in the case of system (b), PCDA molecules form a rather loose disk-like association, and the dye molecule penetrates into it in such a way that both its Ph(C=O) head and its long tail protrude into the surrounding water from opposite sides of the cluster (Figure 3c), and the entire surface of **HCur** is only weakly screened by PCDA molecules.

Thus, the enolic form of **HCur** predominates in the mixed vesicles, although the location of **HCur** molecules within the PC- and PCDA-based bilayers is quite different, which is reflected, e.g., in different exposures of the molecules to the hydrated exterior layer. This can be a reason for the different spectral patterns of **HCur** in the PDA- and PC(PS)-based vesicles, although possible translocations of **HCur** molecules within the PDA-bilayer during photopolymerization of **HCur**-PCDA vesicles cannot be excluded. Moreover, the experimentally observed smaller loading extent of **HCur** into PDA- in comparison with PC(PS)-based vesicles agrees with the MD calculations revealing better compatibility of **HCur** with the phospholipids than with PCDA molecules reflected in more close contact of the dye with the former matrix than with the latter environment. 

The planarity of **HCur** molecules can be distorted when their R-substituents are incorporated into the hydrophobic layer of the vesicles, which may blue-shift the emission bands. The deprotonation of the enolic form of curcumine and curcuminoide derivatives is another reason for the changes in their emissive properties [65]. The phase transitions in the mixed bilayers should also be mentioned as the factor triggering a translocation of the dye molecules from the hydrophobic core of the PhL bilayers to their polar periphery, typically manifested by the changes in the dye spectra [66,67]. Finally, the aggregation of mixed vesicles can be considered as another factor affecting the luminescence of the **HCur** molecules included into the vesicular aggregates.

**Figure 4:**
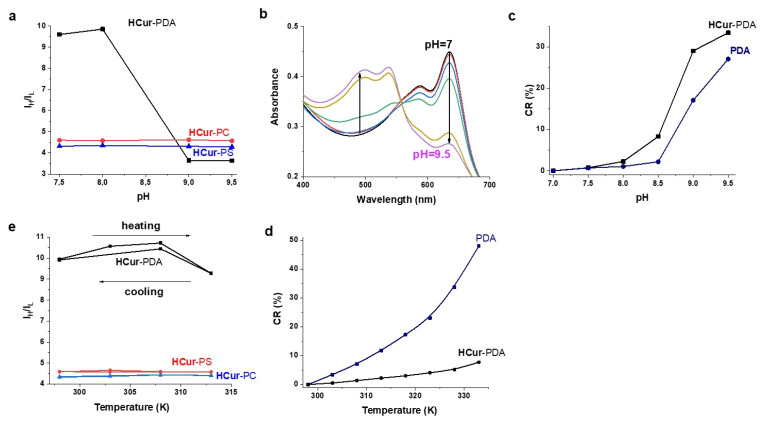
(**a**) I_H_/I_L_ of **HCur**-PC, **HCur**-PS and **HCur**-PDA at different pH values; (**b**) UV-vis spectra of **HCur**-PDA at different pH; (**c**) colorimetric response of **HCur**-PDA and PDA at different pH; (**d**) colorimetric response of **HCur**-PDA and PDA at different temperature (T, K); (**e**) I_H_/I_L_ of **HCur**-PC, **HCur**-PS and **HCur**-PDA at different T.

The incorporation of the dye molecules into the phospholipid vesicles is the reason for the disturbing of the structural ordering in their hydrophobic layers, which can be followed by the enhanced aggregation of the mixed vesicles vs. their pure phospholipid counterparts [68]. The DLS data (Figure 4) measured for the aqueous solutions of the mixed vesicles reveal the average size values, which are greater than the previously reported values of the PC and PDA vesicles [69] and those of the PS vesicles measured in similar conditions (Figure S4). The size and polydispersity characteristics calculated from the DLS data (Table 1) indicate that the incorporation of **HCur** molecules into the PC-, PS- and PDA-based bilayers affects the size and stability of the mixed vesicular aggregates but does not cause significant aggregation of the mixed vesicles. 

### 3.3. Dependence of Spectral Behavior of PDA-HCur, PC-HCur, PS-HCur on External Stimuli (pH, Heating, Metal Ions)

The enolic forms of **HCur** molecules can undergo dissociation in specific pH conditions, followed by the enhancement of the lower energy emission [65]. The lack of detectable changes in the ratios of the intensities of the lower and higher energy transitions of **HCur**-PDA or **HCur**-PC(PS) vesicles under the acidification of their aqueous dispersions to pH values below 7.5 (Figure S5a–c) indicates the insignificant deprotonation of **HCur** molecules in the aforesaid conditions. 

The pH-dependence of the emission was analyzed through the intensity ratios measured for higher- and lower-energy bands (I_h_/I_l_) at 474 and 555 nm, respectively, for **HCur**-PDA, and at 495 and 555 nm, respectively, for **HCur**-PC(PS). The I_h_/I_l_ values at various pHs are plotted in Figure 4a. The results represented in Figure 4a indicate the insignificant changes under the alkalization of **HCur**-PC(PS) aqueous colloids to a pH above 8.5, while the ratios measured for **HCur**-PDA decrease under the alkalization. Similar to the PDA-vesicles themselves [70], the alkalization to pHs above 7.5 triggers the chromatic changes (Figure 4b) peculiar for the blue-to-red transitions of PDA vesicles. It has been already documented that the incorporation of the amphiphilic molecules into the PDA vesicles can stabilize either red [71] or blue forms [36] of the PDA-based backbones. The comparative monitoring of the pH-induced colorimetric changes of **HCur**-PDA and PDA vesicles through the CR% (Colorimetric Response calculated by the Equation (2) in the Supplementary Materials) indicate that both vesicles exhibit the blue-to-red transition at pHs above 8.0, while the transition of **HCur**-PDA becomes detectable at pH > 8.0 and while greater alkalization is required for the similar transition of PDA vesicles (Figure 4c and Figure S4d). 

Heating is another well-known trigger of the blue-to-red transition [72]. Thus, the heating-induced changes of **HCur**-PDA vesicles should be compared with those of PDA itself. The data presented in Figure 4d indicate very poor transitions up to 55 °C, while the PDA vesicles themselves exhibit significant blue-to-red transition in these conditions. 

The fluorescence of **HCur** in the phospholipid vesicles exhibits insignificant changes in the I_h_/I_l_ values under heating (Figure 4e), while changes are detectable under the heating of **HCur**-PDA vesicles. The aforesaid changes can be correlated with the temperature-dependence of both conformational changes of **HCur** in the PDA-vesicles and hydration-induced quenching. Thus, the greater distortions of **HCur** molecules under their incorporation into the PDA-vesicles vs. the PC(PS)-vesicles correlate with the higher sensitivity of **HCur**-PDA in comparison with **HCur**-PC(PS) to the temperature changes.

Coordinative bonds can provide a good basis for the spectral response of **HCur** incorporated into the vesicular aggregates. It is worth noting that the enolic form of **HCur** can coordinate metal ions (Me^2+^) in accordance with the equilibrium (1)

**HCur** + Me^2+^ = [Me**Cur**]**^+^** + H^+^ (1)

**Figure 5:**
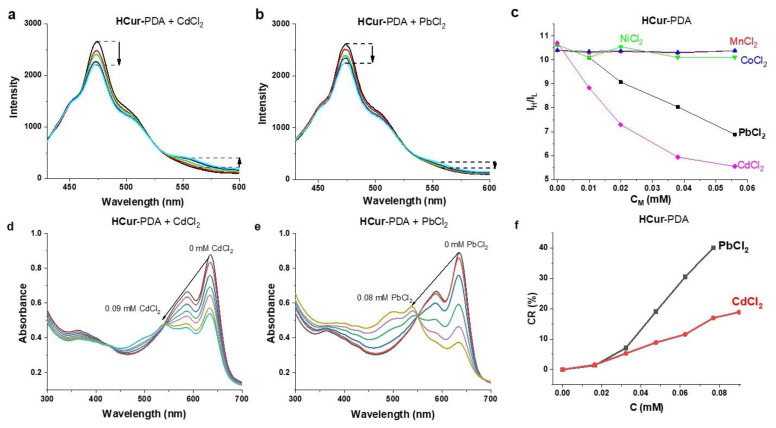
(**a**,**b**) Luminescence spectra of **HCur**-PDA at the concentration of CdCl_2_ and PbCl_2_ varied from 0 to 0.058 mM (pH = 7.5). (**c**) I_H_/I_L_ of **HCur**-PDA vs. concentration of d-metal cations (pH = 7.5): CdCl_2_; PbCl_2_; NiCl_2_; MnCl_2_; CoCl_2_. (**d**,**e**) UV-vis spectra of **HCur**-PDA at pH = 7.5 and different concentration of CdCl_2_ (**d**) and PbCl_2_ (**e**). (**f**) Colorimetric response (CR%) of **HCur**-PDA to CdCl_2_ and PbCl_2_.

However, the spectral response of **HCur** to heavy metal ions is negligible in **HCur**-PC and small in **HCur**-PS (Figure S5), but becomes significant for **HCur**-PDA (Figure 5a,b). This agrees well with the above-mentioned difference in the location of **HCur** molecules within the PC- and PCDA-based bilayers. The concentration-dependent quenching of both the higher (474 nm) and lower (555 nm) energy bands of **HCur**-PDA observed under the increase in concentrations of Co^2+^, Mn^2+^, Ni^2+^ (Figure S6) results from their coordination with the enolate form of **HCur** (Figure S6). The luminescent response of **HCur**-PDA to the growing amounts of Cd^2+^ and Pb^2+^ is manifested by both enhancement of the lower energy and quenching of the higher energy bands (Figure 5a,b). The peculiarity of the luminescent response of **HCur**-PDA to Cd^2+^ and Pb^2+^ ions is demonstrated by the I_h_/I_l_ values (Figure 5c). The values remain practically unchanged in the wide concentration range of Co^2+^, Mn^2+^, Ni^2+^, while the concentration-dependent decrease of I_h_/I_l_ values is observed in the solutions of Cd^2+^ and Pb^2+^ (Figure 5c). The aforesaid peculiarity derives from the increased planarity of the **HCur** molecule under the complex formation with Pb^2+^ and Cd^2+^. Moreover, the I_h_/I_l_ values plotted vs. various concentrations of metal ions reveals the selectivity of the **HCur**-PDA sensor to Cd^2+^ over Pb^2+^ ions (Figure 5c). It is worth noting that the selectivity derives from the conformational changes of **HCur** resulting from its complex formation with Cd^2+^ and Pb^2+^ ions with specific electronic structure (d^10^ and d^10^s^2^ correspondingly). Thus, both nature and lengths of coordination bonds are worth noting among the factors influencing such changes, although all the factors responsible for the sensitivity cannot be specified within the present work scope. 

It is well known that the coordinative binding with Pb^2+^ ions facilitates the blue-to-red transition of the PDA vesicles modified by the additional ligands [73,74,75,76]. The monitoring of the electronic absorption spectra of the **HCur**-PDA vesicles under the growing concentrations of Pb^2+^ and Cd^2+^ (Figure 5d,e) indicates the specific colorimetric response of **HCur**-PDA to Pb^2+^ and Cd^2+^ ions (blue-to-red transition). 

Thus, the aforesaid reveals **HCur**-PDA vesicles as the more sensitive and selective nanosensor to metal ions than **HCur**-PC(PS) vesicles. This correlates with a higher exposure of **HCur** molecules to water in their supramolecular assemblies with PCDA molecules in comparison with the **HCur**-PC assemblies revealed by our computational modeling (vide supra). Moreover, the **HCur**-PDA vesicles demonstrate both fluorescent and colorimetric responses to the heavy metal ions, although there is no correlation between I_h_/I_l_ and CR% values (Figure 5f). In particular, the CR% values can distinguish between Pb^2+^ and Cd^2+^ ions at concentrations above 0.03 mM, while the I_h_/I_l_ values give the selective response to Cd^2+^ over Pb^2+^ ions at concentrations below 0.01 mM. The different selectivity of the chromatic (Pb^2+^) and fluorescent (Cd^2+^) responses of **HCur**-PDA indicates that the latter derives from the complexation of the metal ions with **HCur**, while the former is predominantly affected by the complexation with the PDA platform. 

### 3.4. Sensitivity of PDA-HCur Fluorescence to Polyaminoacids and Proteins

**Figure 6:**
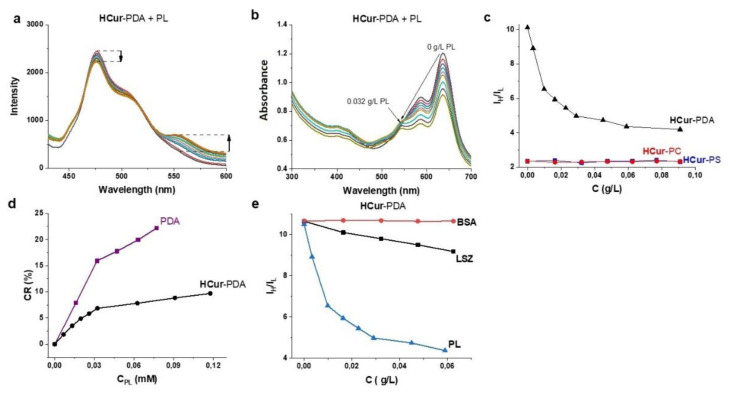
Luminescence (**a**) and electronic absorption spectra (**b**) of **HCur**-PDA at different concentrations of PL (from 0 to 0.091 g/L). (**c**) I_H_/I_L_ of **HCur**-PC, **HCur**-PS and **HCur**-PDA vs. concentration of PL (from 0 to 0.091 g/L). (**d**) Colorimetric response of PDA and **HCur**-PDA to different concentrations of PL. (**e**) I_H_/I_L_ of **HCur**-PDA at different concentration of PL, LSZ and BSA.

The presence of the multiple-surface exposed carboxy/carboxylate groups makes the PDA nanoplatform a convenient basis for the binding of macromolecules through the combined effect of electrostatic attraction and hydrogen bonding. Polylysine (PL) is a good choice to reveal an impact of the latter effect on both colorimetric and fluorescent responses of **HCur**-PC(PS) and **HCur**-PDA vesicular aggregates. PL molecules are well known for their conformational flexibility, being predominantly in α-helical structure at pHs above 10.4 and converting into the coil-like and β-sheet secondary structures at pHs below 8.0 [77]. The spectral response of **HCur**-PDA monitored in a buffer solution at pH 7.5 is evident from both fluorescent and chromatic changes under the increased concentration of PL (Figure 6a,b), which are manifested by the decrease in the I_h_/I_l_ values and the increase in the CR values correspondingly (Figure 6c,d and Figure S7c). It is worth noting that no significant changes in the I_h_/I_l_ values are revealed for **HCur**-PC(PS) under the increased concentration of PL (Figure 6c and Figure S7a,b). 

Thus, similarly to the above-mentioned spectral responses of **HCur**-PDA vesicles to alkalization and Pb(Cd)^2+^ ions, the spectral response to PL reveals the interference between the chromatic and fluorescent changes. In particular, the incorporation of **HCur** is the factor suppressing the chromatic response of **HCur**-PDA to PL in comparison with that of PDA itself (Figure 6d). However, the close-to-linear increase in the CR values covers the concentration range 0–0.04 mM (Figure 6d), while the sharp decrease of the I_h_/I_l_ values within 0.002–0.025 mM of PL comes to the saturation level at 0.02–0.045 mM (Figure 6e and Figure S7d,e). The difference in the concentration ranges required for the chromatic and fluorescent responses of **HCur**-PDA reveals the difference between the binding of PL molecules with the fluorescent (**HCur**) and colorimetric (PDA) sensors. However, the I_h_/I_l_ values of **HCur**-PDA exhibit some decrease under the concentration of PL above 0.045 mM, which correlates with the blue-to-red phase transition of the PDA nanobead triggered by the interaction with PL. This provides one more argument for the interference between the colorimetric and fluorescent responses of **HCur**-PDA to PL.

The results reveal an impact of the surface-exposed amino/ammonium groups of PL molecules on their efficient binding with **HCur**-PDA, which raises a question about a possibility of its fluorescent response to Lysozyme (LSZ) with the net charge at 8e and pI = 10.33 [78]. LSZ does not trigger the chromatic responses of both PDA and **HCur**-PDA vesicles (Figure S6f). The fluorescent response of **HCur**-PDA requires a greater concentration of LSZ in comparison with that of PL. This correlates with the smaller amount of the surface-exposed amino/ammonium groups of LSZ, while the lack of any detectable fluorescent response of **HCur**-PDA to bovine serum albumin (BSA) correlates with its charge (−18e) and pI characteristics (4.7), which are quite different from those of LSZ.

## 4. Conclusions

In summary, the newly synthesized representative of the hemicurcuminoid family bearing a nonyl substituent (**HCur**) forms the supramolecular assemblies with phosphatidylcholine, phosphatidylserine and poly10,12-pentacosadiynoic acid (PCDA) molecules, which allows for the obtaining of mixed vesicular aggregates containing the fluorescent **HCur** through the thin film procedure. The **HCur**-PCDA vesicles were converted into the polymeric ones through photopolymerization. The phospholipid-based vesicles demonstrate the high loading by **HCur** molecules, while the loading extent is much less for the PDA-based vesicles. This correlates with molecular modeling simulations revealing the more favorable encapsulation of **HCur** molecules via their nonyl substituents into the phospholipid-based assemblies, while the PCDA-based assembly provides less efficient screening of **HCur** molecules from the exterior water molecules. 

The single-crystal XRD analysis revealed a high planarity of **HCur** molecules in the solid state, although the planarity distortion of **HCur** may have affected its spectral behavior in the mixed vesicles. The spectral behavior of **HCur** in the PDA-based aggregates differed from that in the phospholipid-based ones. In particular, the fluorescence of **HCur** in the PDA-based vesicles demonstrated much greater sensitivity to the external stimuli (temperature changing from 293 to 313 K and the pH increase from 7.5 to 9.5) than the fluorescence of **HCur**-PC(PS) vesicles. This highlights an impact of greater exposure of **HCur** to a bulk of solution on the sensitivity of its fluorescence to the external stimuli. 

It is also worth noting that **HCur**-PDA is introduced as the dual fluorescent-chromatic nanosensor. The difference in the sensitivity and selectivity of the fluorescent and chromatic responses of **HCur**-PDA indicates that they are driven by the substrate–**HCur** and substrate–PDA interactions correspondingly. Thus, the fluorescent response of **HCur**-PDA vesicles to both heavy metal ions (Pb^2+^ and Cd^2+^) and polylysine is more sensitive than the colorimetric response of the vesicles, although both responses interfere with each other. This highlights both the molecular structure of **HCur** and its supramolecular package into the different vesicular aggregates as the key factors responsible for the sensing ability towards the heavy metal ions and polylysine. However, further modifications on both molecular and supramolecular levels are required to develop a sensor able to recognize protein molecules. 

## Figures and Tables

**Table 1:** Size distribution by volume and intensity of **HCur**-PC (1), **HCur**-PS (2) and **HCur**-PDA (3) at pH = 8.1.

	PdI	Size by Intensity (d_I_, nm)	Size by Volume (d_I_, nm)
**HCur**-PC	0.430	47 ± 8 265 ± 88	43 ± 9 274 ± 99
**HCur**-PS	0.594	125 ± 63	66 ± 49
**HCur**-PDA	0.450	271 ± 100	283 ± 115

## Supplementary Materials

The following supporting information can be downloaded at: https://www.mdpi.com/article/10.3390/polym15030714/s1: Table S1. 1H and 13C chemical shiftsa (ppm) and spin-spin coupling constants (Hz) observed for the enol form of compounds HCur-BF2 and HCur in DMSO-d6 at 303K; Table S2. Experimental crystallographic data for compound 3; Figure S1 HRMS (ESI) of **HCur** (C_26_H_32_O_3_), *m/z*: 393.2420, [M+H]^+^, calcd for C_26_H_33_O_3_ 393.2424; Figure S2 a - UV-vis spectra of HCur at different concentration. b - I_400_ of UV-vis spectra of **HCur** at different concentration. c - UV-vis spectra of residual amount of **HCur** after synthesise of **PC-HCur**, PS**-HCur** and PCDA**-HCur**. (d) – UV-vis spectra of **HCur** in CHCl_3_ at different time of UV-irridiation (254nm); Figure S3 a,b - Emission and excitation spectra of HCur in DMF (emission 470 nm, excitation 390 nm). c,d - Emission and excitation spectra of HCur in CHCl_3_ (emission 460 nm, excitation 340 nm); Figure S4 Size distribution by Volume (red line) and by intensity (black line) of PDA-HCur(a), PC-HCur (b) and PS-HCur (c): a,b,c – at pH = 8.1; d,e,f – at pH = 3.5. g,h,I – size distribution of bilayers aggregates of PC (g), PS(h) and PDA(i); Figure S5. Luminescence spectra of PC-HCur(a), PS-HCur(b) and PDA-HCur(c) at different pHs. d – UV-vis spectra of PDA at different pHs; Figure S6. Luminescence spectra of PDA-HCur in presence of different concentration of CuCl_2_ (a), MnCl_2_ (b) and NiCl_2_ (c); Figure S7. Luminescence spectra of **HCur**-PC(a) ab **HCur**-PS(b) and **HCur**-PDA(3) vs concentration of PL. c – UV-vis spectra of PDA at different concentration of PL. d,e – Luminescence spectra of **HCur**-PDA at different concentration of BSA and LSZ.
