# Supplementary material for: Supramolecular Optimization of Sensory Function of a Hemicurcuminoid through Its Incorporation into Phospholipid and Polymeric Polydiacetylenic Vesicles: Experimental and Computational Insight
Source: Polymers (Basel). 2023 Jan 31;15(3):714. doi: 10.3390/polym15030714 (PMC9920781; doi:10.3390/polym15030714)
Supplement: Supplementary file 1 [file polymers-15-00714-s001.zip › polymers-2123070-supplementary.pdf]

Supramolecular optimization of sensory function of a hemicurcuminoid through its incorporation into phospholipid and polymeric polydiacetylenic vesicles: experimental and computational insight.

B.S. Akhmadeev[a], O.O. Retyunskaya[b], S.N. Podyachev[a], S.A. Katsyuba[a], A.T. Gubaidullin, S. N. Sudakova[a], V.V. Syakaev[a], V.M. Babaev[a],O. G. Sinyashin[a], A.R. Mustafina[a]

[a] *Arbuzov Institute of Organic and Physical Chemistry, FRC Kazan Scientific Center of RAS, 8 Arbuzov st., 420088 Kazan, Russian Federation*

[b] *Kazan (Volga region) Federal University, Kremlyovskaya str., 18, 420008, Kazan, Russia*

**Corresponding author*:

E-mail address: bulat_ahmadeev@mail.ru

Experimental section 1

Methods 2

Synthesis of (E)-5-(4-nonyloxyphenyl)-1-phenylpent-4-ene-1,3-dione-difluoroboron adduct (HCur-BF_2) 3

Synthesis of (E)-5-(4-nonyloxyphenyl)-1-phenylpent-4-ene-1,3-dione (HCur). 3

Table S1 3

Quantitative experimental evaluation of the molar ratio (eq. 1) 4

Figure S1 5

Table S2 5

Figure S2 6

Figure S3 7

Colorimetric Response (eq. 2) 7

Figure S4 8

Figure S5 8

Figure S6 9

Methods

Dynamic light scattering (DLS) measurements were performed by means of the Malvern Mastersize 2000 particle analyzer. A He–Ne laser operating at 633 nm wavelength and emitting vertically polarized lightwas used as a light source. The measured autocorrelation functions were analyzed by Malvern DTS software and the second-order cumulant expansion methods. The effective hydrodynamic radius (RH) was calculated by the Einstein–Stokes relation from the first cumulant: D = kBT/6πηRH, where D is the diffusion coefficient, kB is the Boltzmann constant, T is the absolute temperature, and η is the viscosity. The diffusion coefficient was measured at least three times for each sample. The average error in these experiments is approximately 4%.

The steady-state emission spectra of terbium complexes PDA-H_4L and PDA-H_4L' were recorded on spectrofluorimeters FL3-221-NIR (Horiba Jobin Yvon) and Hitachi F-7100 at 330 nm excitation wavelength.

UV-Vis spectra were recorded on spectrophotometer Specord 50 plus (Analytic Jena). Vesicles were precipitated in a centrifuge MPW-351R at 15000 rpm and 4 ^{o}C. All NMR experiments were performed on a Bruker AVANCE-500 spectrometer.

Microanalyses of C and H were carried out with a EuroVector CHNS-O Elemental Analyser EA3000. Melting points of compounds were measured with a Boetius hotstage apparatus. MALDI mass spectra were detected on a Bruker Ultraflex III MALDI-TOF/TOF mass spectrometer. NMR experiments were performed on a Bruker AVANCE-600 spectrometer at 303K equipped of a 5 mm diameter broadband probe head working at 600.13 MHz in ^{1}H and 150.864 MHz in ^{13}C experiments.

The X-ray diffraction data for the crystal of **HCur** were collected on a Bruker D8 Quest single crystal X-ray diffractometer equipped with an Incoatec IμS microfocus source (Mo Kα, λ = 0.71073 Å), a multilayers optics monochromator, and a PHOTON III area detector, in the ω and φ-scan modes at 108(2) K. The frames were integrated with the Bruker SAINT software package using a narrow-frame algorithm. Data were corrected for absorption effects using the Multi-Scan method by SADABS program [36]. The crystal data, data collection, and the refinement parameters are given in Table S1. The structures were solved by direct method using SHELXS and refined by the full matrix least-squares using SHELXTL programs [37]. All non-hydrogen atoms were refined anisotropically. The position of the hydrogen atom of hydroxyl group was determined based on the electronic density distribution and was refined isotropically. All other hydrogen atoms were placed in idealized positions and constrained to ride on their parent atoms. Data collection: images were indexed and integrated using the APEX3 data reduction package [38]. All calculations were performed on PC using WinGX suit of programs [39]. Mercury program package [40] was used for figures preparation.

Synthesis of HCur

(E)-5-(4-nonyloxyphenyl)-1-phenylpent-4-ene-1,3-dione-difluoroboron adduct ($HCur-BF_2$). Benzoylacetone-difluoroboron adduct (4.76 mM, 1 g) in dry AcOEt (40ml) mixed with 4-nonyloxybenzaldehyde (5.24 mM,1.3 ml) and tributyl borate (7.14 mM,1.90 ml) was stirred in argon atmosphere during 0.5 h at 60-70°C. The stirring was continued for 0.5 h with the slow addition of 1,6-diaminohexane (2.38 mM 0.28 g) in AcOEt (10 ml). After stirring of the solution for overnight at 60-70°C, cold water (40ml) was added to the reaction mixture and again stirred for 0.5 h. Then layers were separated and water layer was extracted with AcOEt (20 ml). Organic layers were combined and washed several times with water. Then after drying under $MgSO_4$, the AcOEt was removed, the residue was twice washed by diethyl ester (20 ml), filtered off and dried in *vacuo* giving the light orange product **$HCur-BF_2$** (0.58g) with 30% yield. Mp. 139 ºC. Anal. calcd for $C_{26}H_{31}B_1F_2O_3$ (440.34): C, 70.92; H, 7.10. Found: C, 70.79; H, 6.92. Mass spectrum (MALDI-TOF): m/z: = 421.3 $[M-F]^+$, 463.3 $[M+Na]^+$, 479.3 $[M+K]^+$.

(E)-5-(4-nonyloxyphenyl)-1-phenylpent-4-ene-1,3-dione (HCur). To hydrolyze the product **2**, the solution of MeOH (15ml) and TEA (0.09ml) was added to **$HCur-BF_2$** (0.45mM, 0.2g). The mixture was stirring under reflux during 4 h. Then 2/3 of the MeOH volume was distilled off and H_2O (20ml) was added. After stirring, a yellow solid was filtered off and dried, giving 0.12g of **HCur** with the yield 67%. Mp. 91 ºC. Anal. calcd for $C_{26}H_{32}O_3$ (392.54): C, 79.56; H, 8.22. Found: C, 79.36; H, 7.94. Mass spectrum (MALDI-TOF): m/z: = 393.2 $[M+H]^+$, 415.1 $[M+Na]^+$.

Table S1. ^1H and ^{13}C chemical shifts[a] (ppm) and spin-spin coupling constants (Hz) observed for the enol form of compounds **$HCur-BF_2$** and **HCur** in DMSO-d^6 at 303K.

atom	compound			
	**$HCur-BF_2$**		**HCur**	
	^1H	^{13}C	^1H	^{13}C
1	7.654 t, ^3J=7.6	134.9	7.640 m	132.8
2	7.526 t, ^3J=7.6	129.2	7.561 t, ^3J = 7.3	128.8
3	8.079 d, ^3J=7.6	128.8	8.009 d, ^3J = 7.3	127.1
4		132.4		135.4
5		181.5		187.3
6	6.607 s	97.6	6.754 s	97.0
7		181.4		181.0
7’			16.471 br (OH_{enol})	
8	6.682 d, ^3J=15.5	118.0	6.830 d, ^3J = 15.9	121.0
9	8.092 d, ^3J=15.5	148.40	7.679 d, ^3J = 15.9	140.0
10		126.7		127.1

11	7.594 d, ^{3}J=8.8	131.7	7.662 d, ^{3}J = 8.3	130.0
12	6.953 d, ^{3}J=8.8	115.5	7.004 d, ^{3}J = 8.3	115.0
13		163.0		160.6
14	4.034 t, ^{3}J=6.6	68.6	4.022 t, ^{3}J = 6.4	67.7
15-21	1.819 - 1.309 m	32.1- 22.9	1.264 - 1.716 m	31.2-22.0
22	0.901 t, ^{3}J=6.9	14.3	0.858 t, ^{3}J = 6.3	13.9

[a] Numbering according to Scheme 1

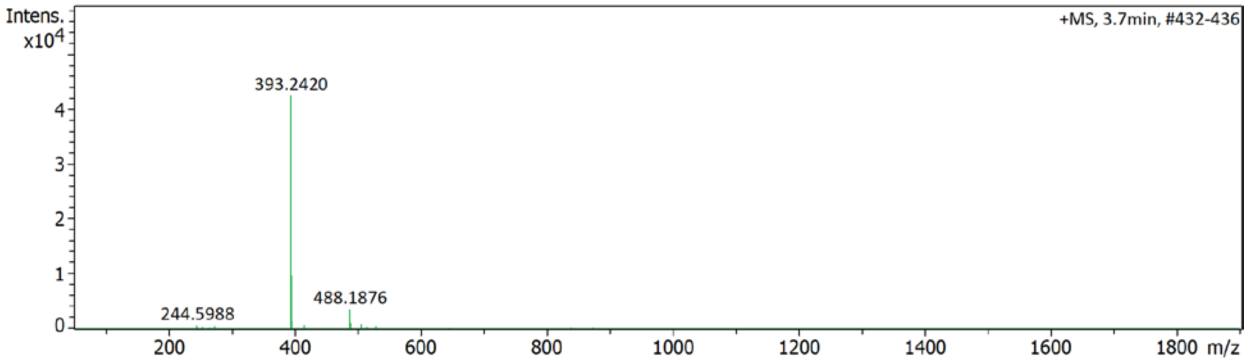

Fig. S1 HRMS (ESI) of **HCur** ($C_{26}H_{32}O_3$), *m/z*: 393.2420, $[M+H]^+$, calcd for $C_{26}H_{33}O_3$ 393.2424.

Samples were analysed using an Impact II («Bruker Daltonik GmbH», Germany) mass spectrometer with an Elute UHPLC («Bruker Daltonik GmbH», Germany) LC system. The column used was a YMC-Triart C18 (50X2,0 mm; 3 µm). Elution solvents used were Milli-Q water + 0.1% FA (A) and HPLC-grade acetonitrile + 0.1% FA (B) and elution gradient was the following: 0 min at 50% B, 2 min at 5% B, 4 min at 5% B, 4.1 min at 50% B, 6 min at 50% B with a flow rate of 0.3 mL/min. Analytes were ionized by electrospray in positive polarity. ESI conditions were set with the capillary temperature at 220 °C, capillary voltage at 4.5 kV, and a sheath gas flow rate of 6 L/min. Measurements were made in the range *m*/*z* 50-1900. The solution of analyte in acetonitrile at a concentration of 3 mkg/ml and aliquots of 2 µL was used. The solution of sodium iodide in Milli-Q water (200 g/L) was used as a calibrant. The relative error in determining the masses no more than 1.0 ppm. The *m*/*z* values of monoisotopic ions are given in the descriptions. For instrument control and data acquiring the otofControl software (Bruker Daltonik GmbH, Version 5.2) was used. Data processing was performed by DataAnalysis software (Bruker Daltonik GmbH, Version 5.3).

Quantitative experimental evaluation of the molar ratio.

The incorporation of **HCur** into PC, PS or PCDA aggregates under the thin film hydration step was quantitatively evaluated by measuring the residual amounts after the hydration step. Therefore, the flask after the hydration step and evacuation of the aqueous colloids was dried under air. The residual amount of HCur was dissolved in 9 ml of $CHCl_3$ with further recording of the UV-Vis spectra (Fig S1) of the obtained solutions. The absorption intensities at 400 nm of the recorded spectra were quantitatively compared with the values evaluated from the spectra recorded at various concentration of HCur in order to determine their residual concentration in the TCM solutions (C^{TCM}). The final concentration of the H_4L (H_4L') in 16 mL of the PCDA-H_4L (H_4L') aqueous colloids was determined by the formula:

$$C(final) = C^{initial} - C^{TCM} \frac{V^{TCM}}{V^{Water}}, (eq. 1)$$

$$where\ C^{initial} = 0.5\ mM, V^{Water} = 16\ ml,\ V^{TCM} = 9 ml,$$

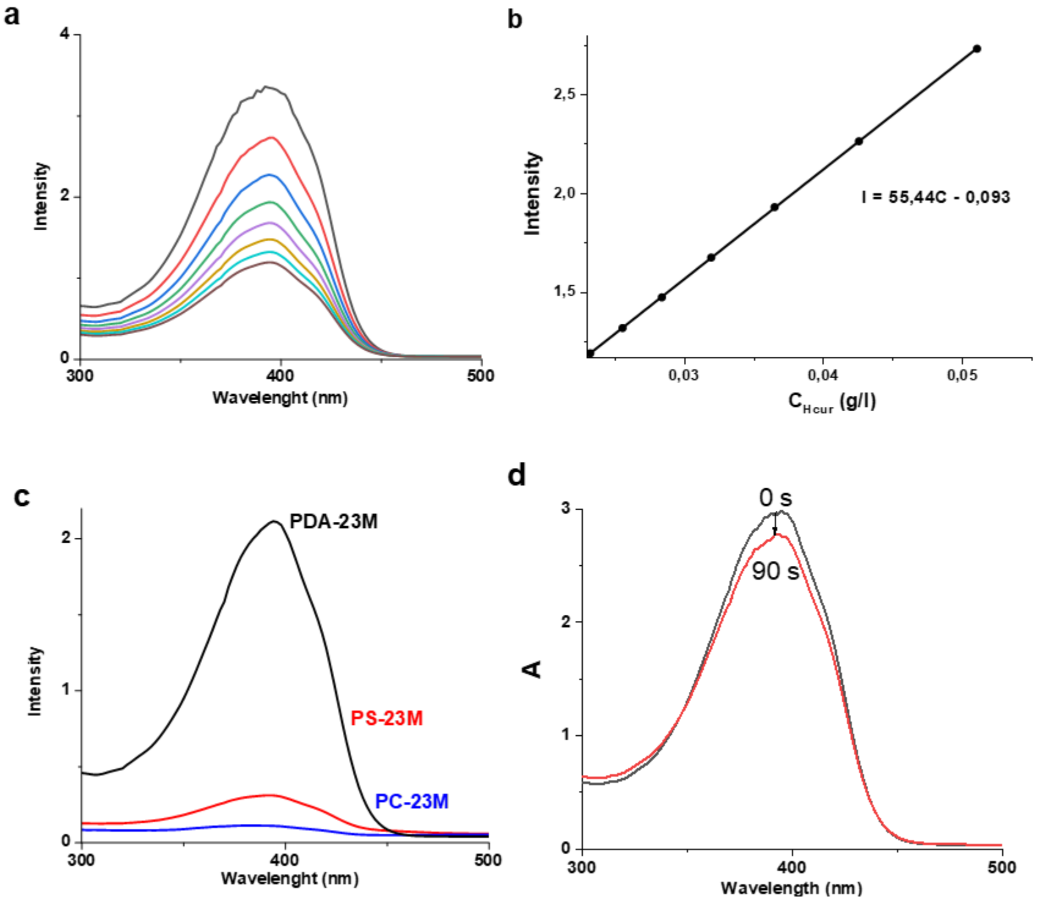

Fig. S2 a - UV-vis spectra of HCur at different concentration. b - I_{400} of UV-vis spectra of **HCur** at different concentration. c - UV-vis spectra of residual amount of **HCur** after synthesise of **PC-HCur**, PS-**HCur** and PCDA-**HCur**. (d) – UV-vis spectra of **HCur** in $CHCl_3$ at different time of UV-irridiation (254nm).

Table S2. Experimental crystallographic data for compound **3**.

Formula	$\mathbf{C_{26}H_{32}O_3}$
Crystal class	*Monoclinic*
Space group	*C* 2/c
Z, Z´	8, 1
Cell parameters	*a* = 14.5860(10) Å,<br>*b* = 5.5882(3) Å,<br>*c* = 53.213(3) Å,<br>β = 94.730(2) °
V, Å^3	4322.6(5) Å^3
M (g/mol)	392.52
T, K	108(2)
Size, mm	0.050 x 0.396 x 0.576
F(000)	1696
ρ_{calc} g/cm^3	1.206
μ, cm^{-1}	0.77

θ, deg	1.536 ≤ θ ≤ 30.941
Refl. meas.	52311
Independ/ Rint	6615 / 0.1385
Completeness	96.7 %
Param./restr	271 / 0
Refl. [I>2σ(*I*)]	4972
R_1 / wR_2	0.0602 / 0.1571
R_1/wR_2 (all refl.)	0.0813/ 0.1738
Goodness-of-fit	1.044
ρ_{max}/ρ_{min} (eÅ^{-3})	0.482 / -0.300

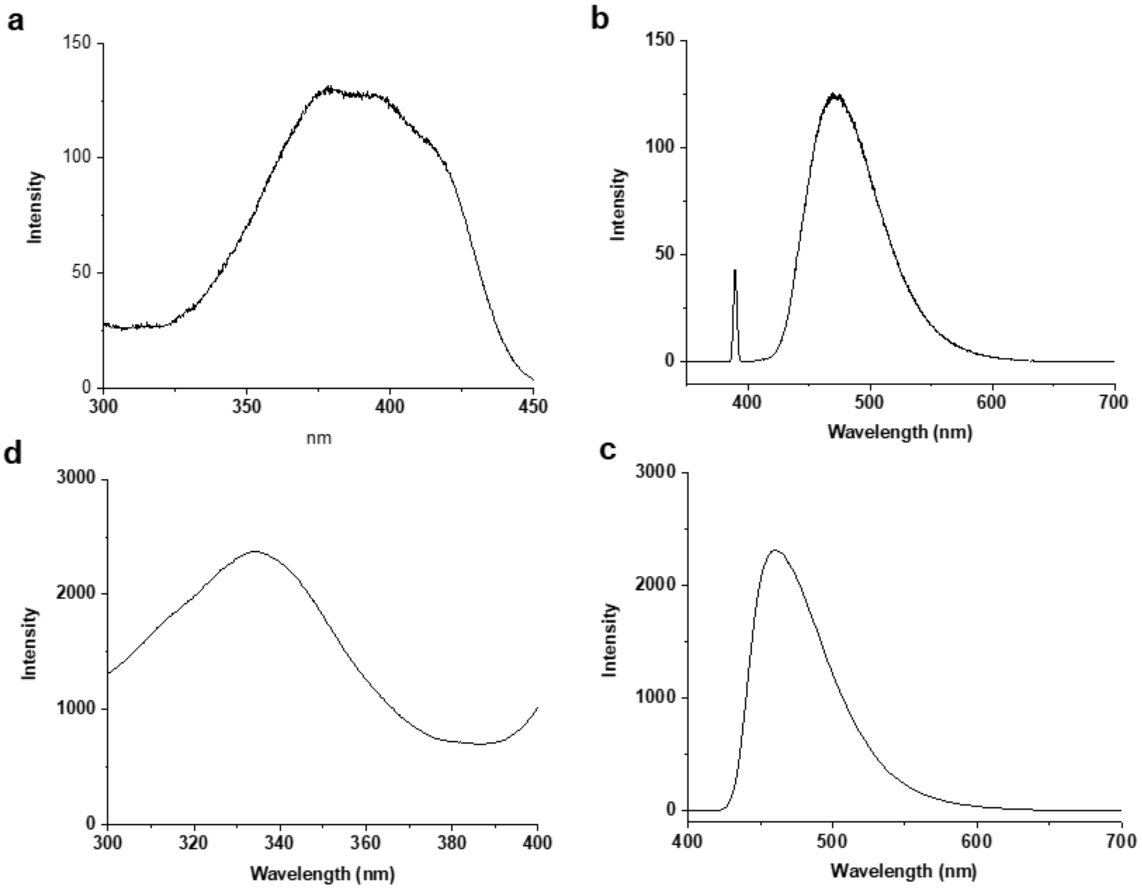

Fig. S3 a,b - Emission and excitation spectra of HCur in DMF (emission 470 nm, excitation 390 nm). c,d - Emission and excitation spectra of HCur in $CHCl_3$ (emission 460 nm, excitation 340 nm).

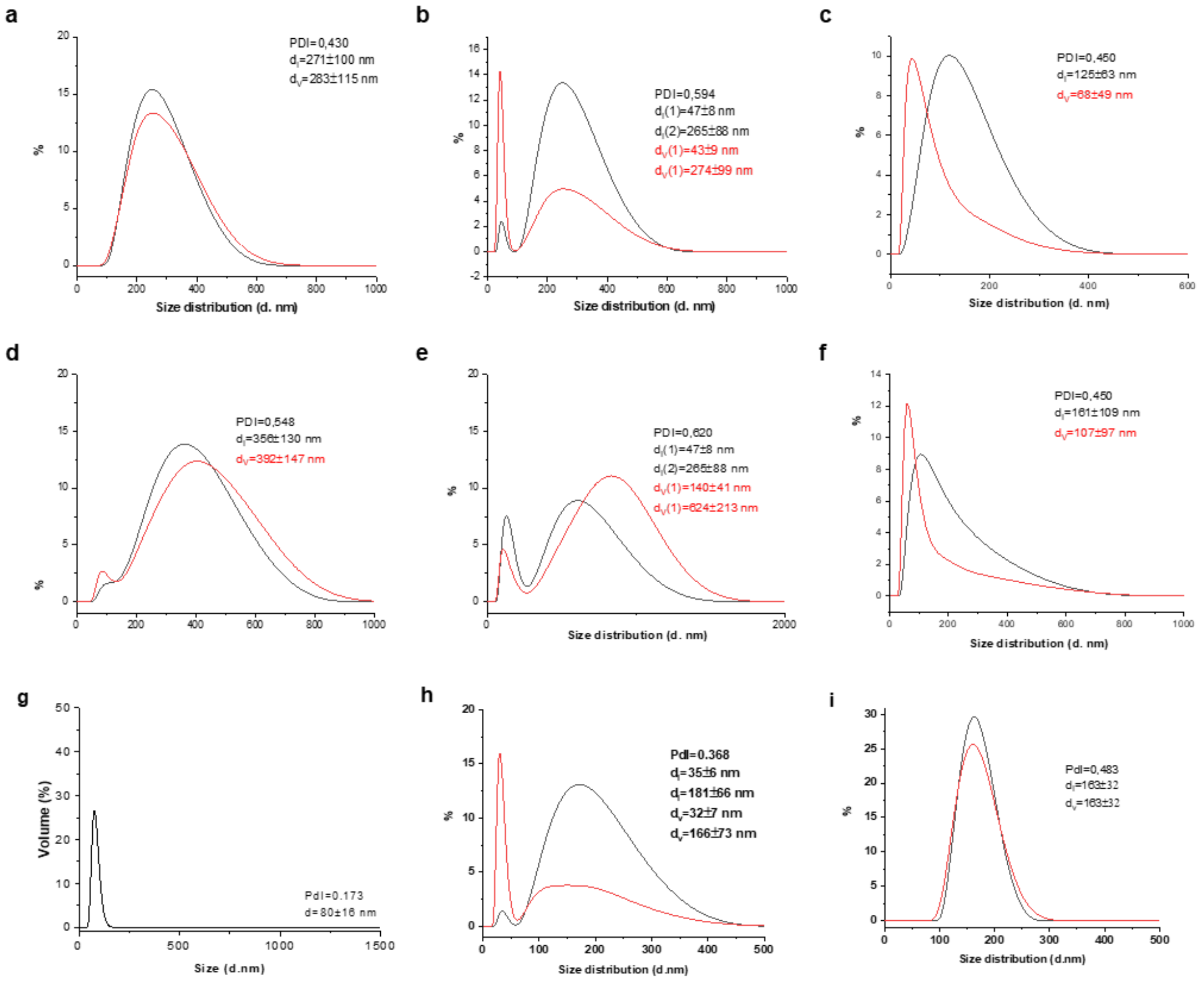

Fig. S4 Size distribution by Volume (red line) and by intensity (black line) of PDA-HCur(a), PC-HCur (b) and PS-HCur (c): a,b,c – at pH=8.1; d,e,f – at pH=3.5. g,h,I – size distribution of bilayers aggregates of PC (g), PS(h) and PDA(i).

Colorimetric Response (%)

$$CR(\%) = \frac{PB_0 - PB_f}{PB_0} * 100, (eq.2)$$

$$PB = \frac{A_{blue}}{(A_{blue}+A_{red})}$$

A_{blue-} is the absorbance at either the blue component (≈650 nm)
A_{red-} is the absorbance at either the red component (≈540 nm)

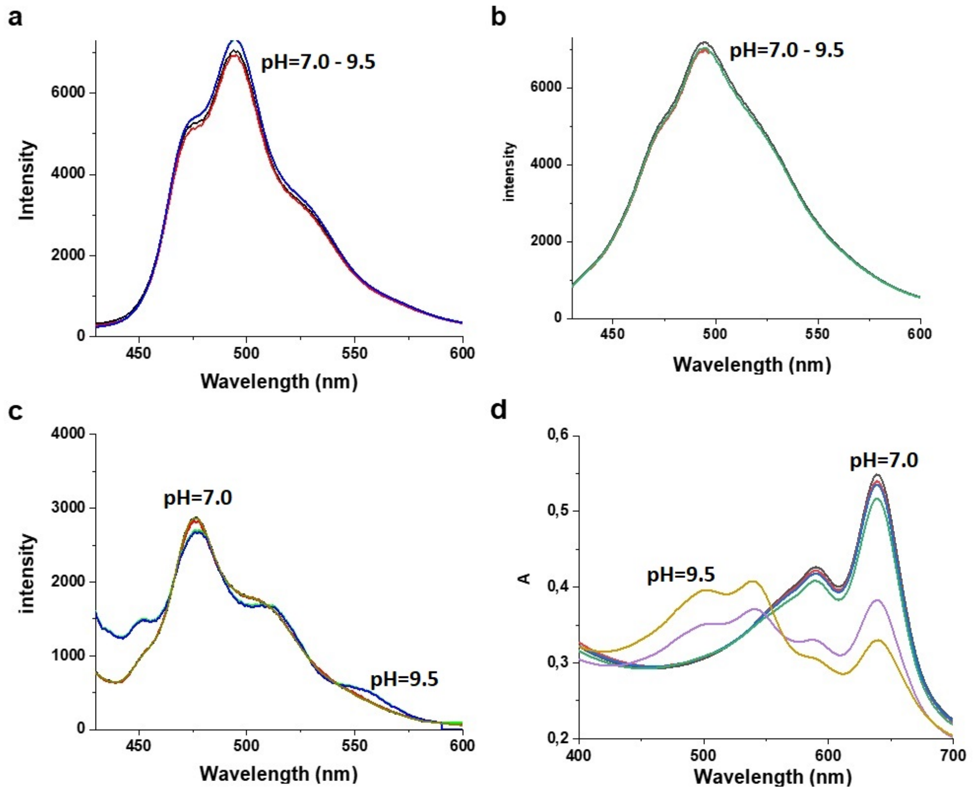

Fig S5. Luminescence spectra of PC-HCur(a), PS-HCur(b) and PDA-HCur(c) at different pHs. d – UV-vis spectra of PDA at different pHs.

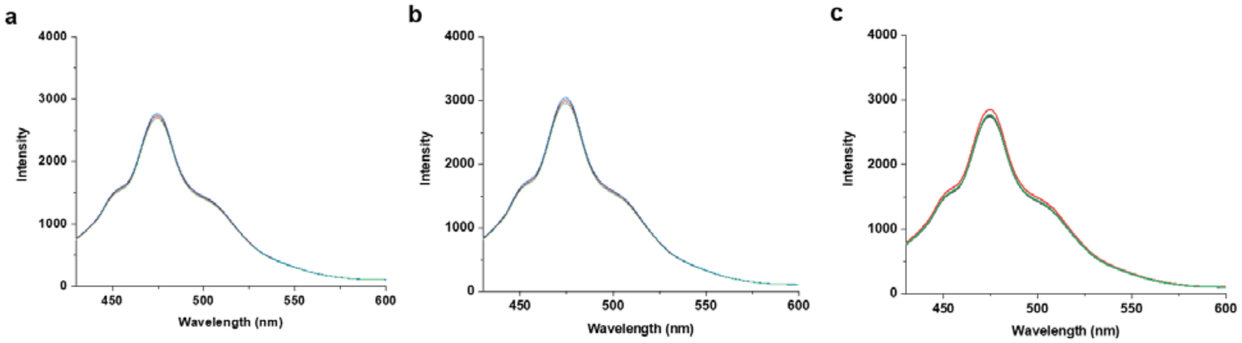

Fig. S6. Luminescence spectra of PDA-HCur in presence of different concentration of $CuCl_2$ (a), $MnCl_2$ (b) and $NiCl_2$ (c).

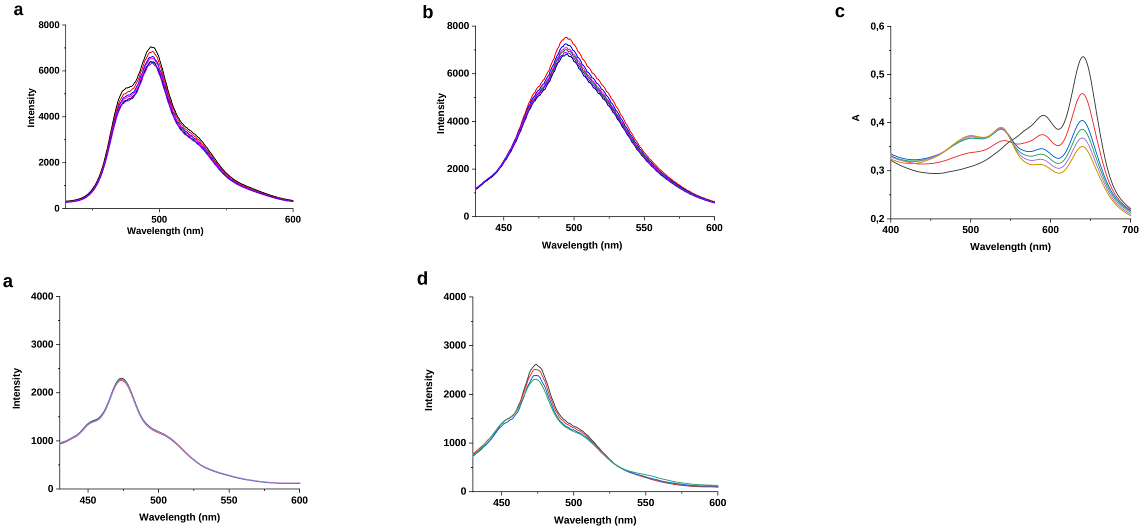

Fig S7. Luminescence spectra of **HCur**-PC(a) ab **HCur**-PS(b) and **HCur**-PDA(3) vs concentration of PL. c – UV-vis spectra of PDA at different concentration of PL. d,e – Luminescence spectra of **HCur**-PDA at different concentration of BSA and LSZ
